# NAD(P)H fluorescence lifetime imaging of live intestinal nematodes reveals metabolic crosstalk between parasite and host

**DOI:** 10.1038/s41598-022-10705-y

**Published:** 2022-05-04

**Authors:** Wjatscheslaw Liublin, Sebastian Rausch, Ruth Leben, Randall L. Lindquist, Alexander Fiedler, Juliane Liebeskind, Ingeborg E. Beckers, Anja E. Hauser, Susanne Hartmann, Raluca A. Niesner

**Affiliations:** 1 Biophysical Analytics, Deutsches Rheuma-Forschungszentrum, Berlin, A Leibniz Institute, Berlin, Germany; 2 Dynamic and Functional In Vivo Imaging, Department of Veterinary Medicine, Institute for Veterinary Physiology, Freie Universität, Berlin, Germany; 3 Department of Veterinary Medicine, Institute of Immunology, Freie Universität, Berlin, Germany; 4 Present Address: Department of Nuclear Medicine, Charité – Universitätsmedizin, Berlin, Corporate Member of Freie and Humboldt University, Berlin, Germany; 5 Laboratory for Immune Dynamics, Deutsches Rheuma-Forschungszentrum, Berlin, A Leibniz Institute, Berlin, Germany; 6 Intravital Microscopy and Immune Dynamics, Department of Rheumatology and Clinical Immunology, Charité – Universitätsmedizin, Berlin, Corporate Member of Freie and Humboldt University, Berlin, Germany; 7 Berliner Hochschule für Technik, University of Applied Sciences, Berlin, Germany

**Keywords:** Biological fluorescence, Biophysics, Immunology, Infection

## Abstract

Infections with intestinal nematodes have an equivocal impact: they represent a burden for human health and animal husbandry, but, at the same time, may ameliorate auto-immune diseases due to the immunomodulatory effect of the parasites. Thus, it is key to understand how intestinal nematodes arrive and persist in their luminal niche and interact with the host over long periods of time. One basic mechanism governing parasite and host cellular and tissue functions, metabolism, has largely been neglected in the study of intestinal nematode infections. Here we use NADH (nicotinamide adenine dinucleotide) and NADPH (nicotinamide adenine dinucleotide phosphate) fluorescence lifetime imaging of explanted murine duodenum infected with the natural nematode *Heligmosomoides polygyrus* and define the link between general metabolic activity and possible metabolic pathways in parasite and host tissue, during acute infection. In both healthy and infected host intestine, energy is effectively produced, mainly via metabolic pathways resembling oxidative phosphorylation/aerobic glycolysis features. In contrast, the nematodes shift their energy production from balanced fast anaerobic glycolysis-like and effective oxidative phosphorylation-like metabolic pathways, towards mainly anaerobic glycolysis-like pathways, back to oxidative phosphorylation/aerobic glycolysis-like pathways during their different life cycle phases in the submucosa versus the intestinal lumen. Additionally, we found an increased NADPH oxidase (NOX) enzymes-dependent oxidative burst in infected intestinal host tissue as compared to healthy tissue, which was mirrored by a similar defense reaction in the parasites. We expect that, the here presented application of NAD(P)H-FLIM in live tissues constitutes a unique tool to study possible shifts between metabolic pathways in host-parasite crosstalk, in various parasitic intestinal infections.

## Introduction

Infections with gastrointestinal nematodes constitute a major burden for the global health of humans and animals alike. Approximately 25% of the world’s population is affected, especially in the tropical and subtropical regions^1,2^. In animal husbandry, nematode infections are widely distributed and lead to huge economic losses in farming. *Heligmosomoides polygyrus* is a natural parasite of the gastrointestinal tract of mice and a widely used model to study intestinal nematode infections. When *H. polygyrus* larvae are taken up orally, they invade the intestinal submucosa, develop to L4 larvae and further into adult worms, which reenter the intestinal lumen^3^. The adult worms move to the proximal part of the small intestine, where they persist and induce chronic infection. Whereas the modulatory effects of the nematode infection on the host immune system and on the microbial environment have been investigated^4–11^, less it is known about the enzymatic activity, energetics and metabolism of the nematodes themselves, which govern the way the parasites persist in the intestinal environment. Up to now, information on metabolism of live parasites (e.g. nematodes) in the host intestine was mostly acquired using bulk biochemical approaches, without spatial or temporal specification^12^.

Intravital two-photon microscopy extended by fluorescence lifetime imaging (FLIM) of nicotinamide adenine dinucleotide (NADH) and nicotinamide adenine dinucleotide phosphate (NADPH), hereafter NAD(P)H, provides one of the most versatile tools to investigate NAD(P)H-dependent metabolic activity in live tissues, with cellular or even subcellular resolution^13–15^. The coenzymes NADH and NADPH are able to bind to diverse enzymes and thereby participate in various metabolic pathways and in reductive biosyntheses within cells^16–20^. The fluorescence lifetimes of both unbound NADH and unbound NADPH lay at ≈ 450 ps^21,22^, both being the average of the fluorescence lifetime over two folding states of each type of coenzyme molecule (≈ 200 ps and ≈ 700 ps)^23^. When bound to enzymes, the fluorescence lifetime of NAD(P)H becomes markedly longer (≈ 2000 ps), and it strongly depends on its enzymatic binding site. Taking into account the abundance of NAD(P)H-dependent enzymes in living cells (mostly conserved across species), we previously developed a systematic frame work to evaluate NAD(P)H-FLIM data^24^. After transforming the time-domain FLIM data in the phase domain via the phasor approach, we first differentiate between free and enzyme-bound NAD(P)H in a two-component manner, defining for each data point (each pixel in an image) the ratio of free to bound NAD(P)H in percent and, hence, generating a metabolic activity map. In a second step, we generate for each pixel a vector representing the vectorial difference between the phase vector of free NAD(P)H and the phase vector of entirely enzyme-bound NAD(P)H, located on the phasor half-circle. This vector is then compared to the reference vectors of the most abundant NAD(P)H-dependent enzymes (RNASeq data^24^) and, based on the similarity to these vectors, probabilities for the binding to the specific enzymes are calculated. We assume that, since there are more than 370 NAD(P)H-dependent enzymes, only the most abundant enzymes will have an impact on the resulting fluorescence lifetime of NAD(P)H and will preferentially bind to the coenzymes. Thereby, the sum of probabilities per pixel equals 100%. Whereas resolving between enzymes with similar NAD(P)H binding sites (similar fluorescence lifetimes of NAD(P)H) is not possible, differentiating between enzymes mainly responsible for metabolic pathways resembling anaerobic glycolysis features (especially LDH) and those mainly responsible those pathways resembling oxidative phosphorylation/aerobic glycolysis features (among them, IDH and PDH), can be easily achieved, both in phase and in time domain, due to the great difference in NAD(P)H fluorescence lifetime: 1600 ps vs. 2100 ps/2500 ps. Whereas previously the preferential energy supply via anaerobic glycolysis has been explained in NAD(P)H-FLIM data solely by more unbound coenzymes leading to a shortening of the mean fluorescence lifetime^25,26^, it has been shown that the glucose uptake under anaerobic conditions is increased, more glucose transporters such as GLUT1 uniporter are expressed^27^ and, thus, the energy production involving NADH is not diminished (i.e. more free, less enzyme-bound coenzymes) but rather takes place in the cytosol, with involvement of LDH. Moreover, binding to NADPH oxidases, responsible for oxidative burst, can also be easily identified due to the specific location of these enzymes within the cell, i.e. they are membrane bound, and due to the particularly long NADPH fluorescence lifetime, i.e. 3650 ps.

Two-photon laser-scanning microscopy^28^, characterized by large imaging depth and optical sectioning in the tissues of living animals, provides information on cellular dynamics and orchestration during diverse pathophysiological situations. In order to account for cellular and tissue functionality, live tissue or in vivo metabolic imaging, is required for a full understanding of these pathophysiological phenomena, besides the analysis of cellular dynamics and communication. This has previously been achieved by combining NAD(P)H FLIM with two-photon microscopy in mammals, especially in mice^29^. However, there is only a single study using the free-living nematode *Caenorhabditis elegans* by means of NAD(P)H-FLIM showing specific metabolic pathways in various tissue regions of the worm, associated with the respective cellular differentiation stage^30^. The genuine environment of *H. polygyrus* is the murine gut, in contrast to *C. elegans*, which lives in the soil. In order to characterize the metabolism of *H. polygyrus*, governing locomotion, persistence and defense in the gut of the host, we aimed to establish NAD(P)H-FLIM of the parasite in the murine gut.

The fluorescence lifetime in general gives information about the molecular environment of the investigated fluorophore. As such, fluorescence lifetime imaging has been used to quantify different vital parameters in cells and tissues, e.g. ionic strength, calcium levels, pH values, protein folding and cleavage using Foerster Resonant Energy Transfer (FRET), temperature or viscosity^16,26,31–42^. The fluorescence of the ubiquitous co-enzymes NADH and NADPH has been extensively used for FLIM to monitor metabolic activity^41^. The NAD(P)H metabolic activity can be correlated to specific cellular phenotypes and functions, with application in health and different pathologies^29^. While many technologies to perform FLIM have been proposed, both in time and in frequency domain^43–46^, time-correlated single-photon counting has proved to be the most reliable and has found broad application for deep-tissue imaging. Moreover, combining FLIM with spectral information recently boosted our insights into biological processes^47^.

In the current work, we used time-correlated single photon counting by two-photon laser-scanning microscopy to demonstrate the feasibility and benefits of NAD(P)H-FLIM to monitor contributions of various metabolic pathways of the live host tissue and, in parallel, of the intestinal parasite *H. polygyrus*. By applying our systematic evaluation of NAD(P)H-FLIM data^24^, we achieve deep insights into host and parasite metabolism, by correlating contributions of main metabolic pathways relying on shifts in NAD(P)H-dependent enzymatic activity with the general metabolic activity of both the host and the parasite.

## Results

### NAD(P)H fluorescence lifetime imaging of murine intestinal environment

**Figure 1:**
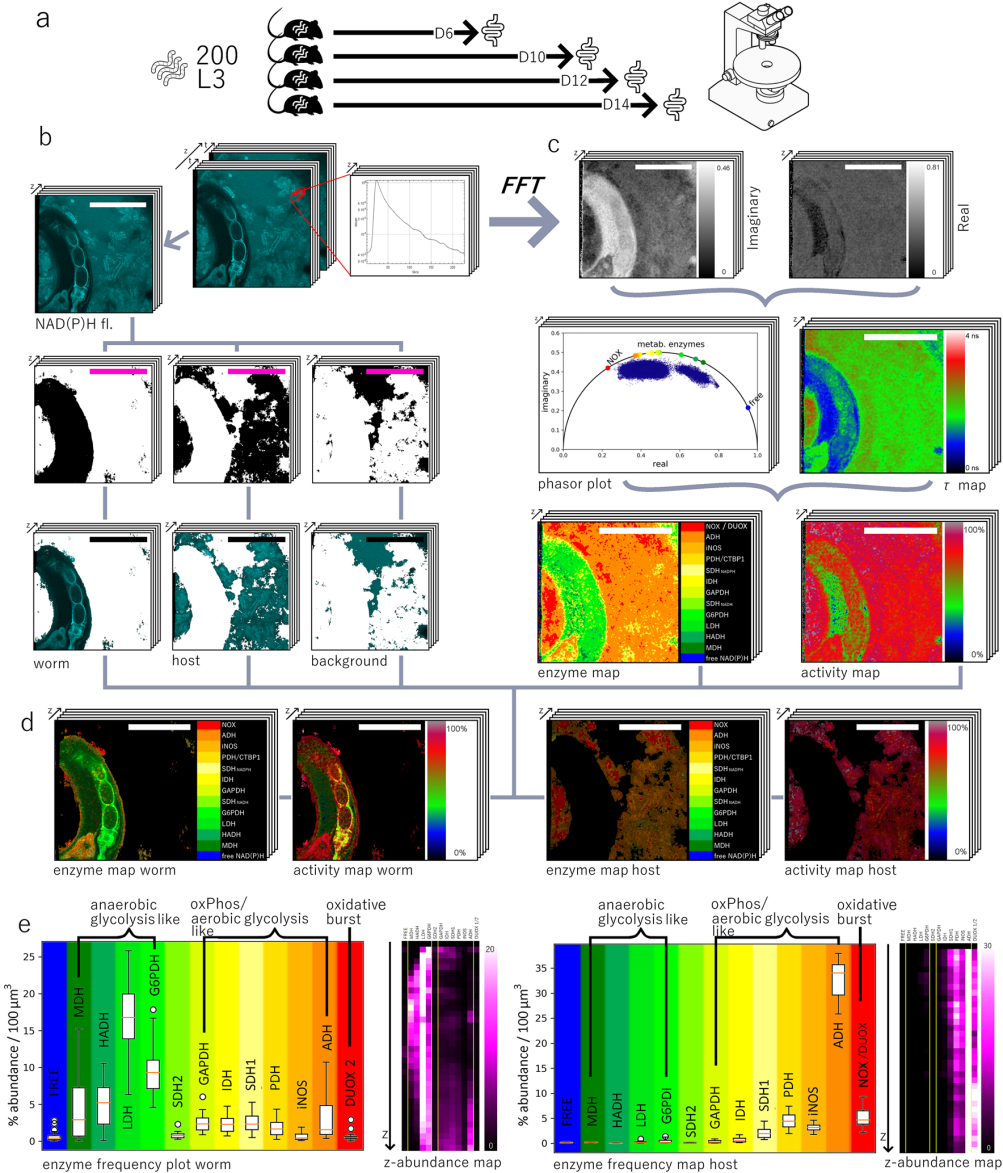
Principle of NAD(P)H fluorescence lifetime imaging of *Heligmosomoides polygyrus* in murine intestinal environment and data evaluation. **(a)** Schematics of experimental setup for infection and imaging. **(b)** Time-resolved NAD(P)H fluorescence images, i.e. for each pixel a fluorescence decay curve as shown is acquired, and NAD(P)H fluorescence sum images generation, typical results of NAD(P)H fluorescence sum image segmentation using the UNet pixel classification algorithm (Ilastik) and mask generation of various entities within the image, here parasite and host tissue. **(c)** Work-flow for phasor-based analysis of NAD(P)H-FLIM data as acquired in **(b)**, including the generation of maps showing the preferential NAD(P)H-dependent enzymatic activity in each pixel (enzyme map) and maps showing the general NAD(P)H metabolic activity, i.e. activity maps. **(d)** Masked enzyme maps and activity maps for parasite and host intestinal tissue, respectively. All data are acquired in a three-dimensional manner, typically 500 × 500 × 100 µm^3^ (505 × 505 × 51 voxel). Scale bar for all images = 250 µm. **(e)** Corresponding pixel frequency graphs for the main NAD(P)H-dependent enzymes in parasite (left side) and host tissue (right side), showing trends towards the main metabolic pathways related to certain enzymatic activities. The purple graphs show the depth dependent frequency of enzyme activity for both parasite and host. Figure generated with Paint.NET, https://www.getpaint.net/download.html.

Using NAD(P)H-FLIM, we analyzed the general metabolic activity and possible enzymatic activity of the nematode *H. polygyrus* and of its natural environment, the luminal side of duodenum in mice. In order to access the intestinal niche of adult *H. polygyrus* nematodes, duodenum of infected C57/Bl6 mice was explanted and cut open to expose its luminal side. Here, the parasites, coiled around intestinal villi, persist over several weeks (Fig. 1a).

The NAD(P)H coenzymes present both in nematodes and in enterocytes and cells of the lamina propria of murine intestinal villi, were also used to visualize worm and host tissue architecture. Based on NAD(P)H fluorescence images, we used a UNet-based pixel classification algorithm to segment the nematodes as well as epithelial layer and lamina propria of the murine intestinal villi and to exclude background regions (Fig. 1b; “Materials and methods” section).

The time-resolved NAD(P)H fluorescence decay acquired in each pixel of the image (Fig. 1c) was recorded by time-correlated single-photon-counting^48,49^ and evaluated using the phasor approach to FLIM^50^, generating the real and imaginary phase maps and the average fluorescence lifetime (τ) map (Fig. 1c). All NAD(P)H-FLIM data considered for further analysis, both in host and in parasites, fulfilled the requirement of a signal-to-noise ratio (SNR) of more than 5, guaranteeing a reliable interpretation of the data according to our previously published approach (Supp. Fig. 1).

Our systematic framework on enzyme-specific analysis of NAD(P)H-FLIM data allows us to assess the general metabolic activity, i.e. the percentage of bound NAD(P)H from the total NAD(P)H—activity map and the presumable preferences in enzymatic activity—enzyme map—in each pixel of the acquired image, both for the worm and for the host tissue (Fig. 1d). High values in the activity map indicate high metabolic activity, whereas low values indicate decreased metabolism, possibly associated with dormancy, low temperature or cell death^51^. The preferential binding of NAD(P)H to most abundant enzymes in the reference enzyme map may be associated with certain metabolic pathways within cells and tissues. Hence, metabolic pathways resembling anaerobic glycolysis features are not necessarily associated only with a reduction of coenzyme binding, i.e. more free NAD(P)H, but also with a preferential activation of cytosolic enzymes, especially lactate dehydrogenase (LDH), as it has been previously shown that under hypoxic conditions cells express more GLUT1 uniporter for an increased uptake of glucose^27^. The activation of mitochondrial enzymes, among them pyruvate dehydrogenase (PDH) or isocitrate dehydrogenase (IDH), leads rather to metabolic pathways resembling oxidative phosphorylation (oxPhos)/aerobic glycolysis, while the activation of NADPH oxidases (NOX/DUOX enzymes) is unequivocally associated with metabolic defense, i.e. via oxidative burst^52^. As emphasized in the introduction, the reference enzyme activation map relies on the abundance of NAD(P)H-dependent enzymes resulting from RNASeq data in mammal cells. This abundance holds true also for *H. polygyrus*^53^, so that we can use the same reference also for the parasites. As we cannot avoid statistical uncertainty in distinguishing the specific activity of enzymes with similar NAD(P)H binding sites, i.e. similar enzyme-bound NAD(P)H fluorescence lifetime and phase vectors, we refer to preferential binding to the enzymes associated with the main metabolic pathways. Concretely, we attribute the phase vectors similar to mitochondrial enzyme-bound NAD(P)H to metabolic pathways resembling the features of oxPhos/aerobic glycolysis, the phase vectors similar to LDH-, HADH- and MDH-bound NAD(P)H to metabolic pathways resembling features of anaerobic glycolysis and the phase vectors similar to NADPH bound to NADPH oxidases to defense mechanisms relying on highly reactive small radicals, with their main representative being the oxidative burst. Thereby, we rely on fluorescence lifetime differences of approx. 1000 ps, as confirmed in other cell types by inhibition of mitochondrial enzymes with rotenone^54^, inhibition of LDH with FX11 and inhibition of NADPH oxidases with AEBSF^21^. Thus, the heterogeneity of dominant metabolic pathways in worm and host, with high axial (z) resolution, can be monitored (Fig. 1e).

### NAD(P)H metabolism of explanted duodenum

**Figure 2:**
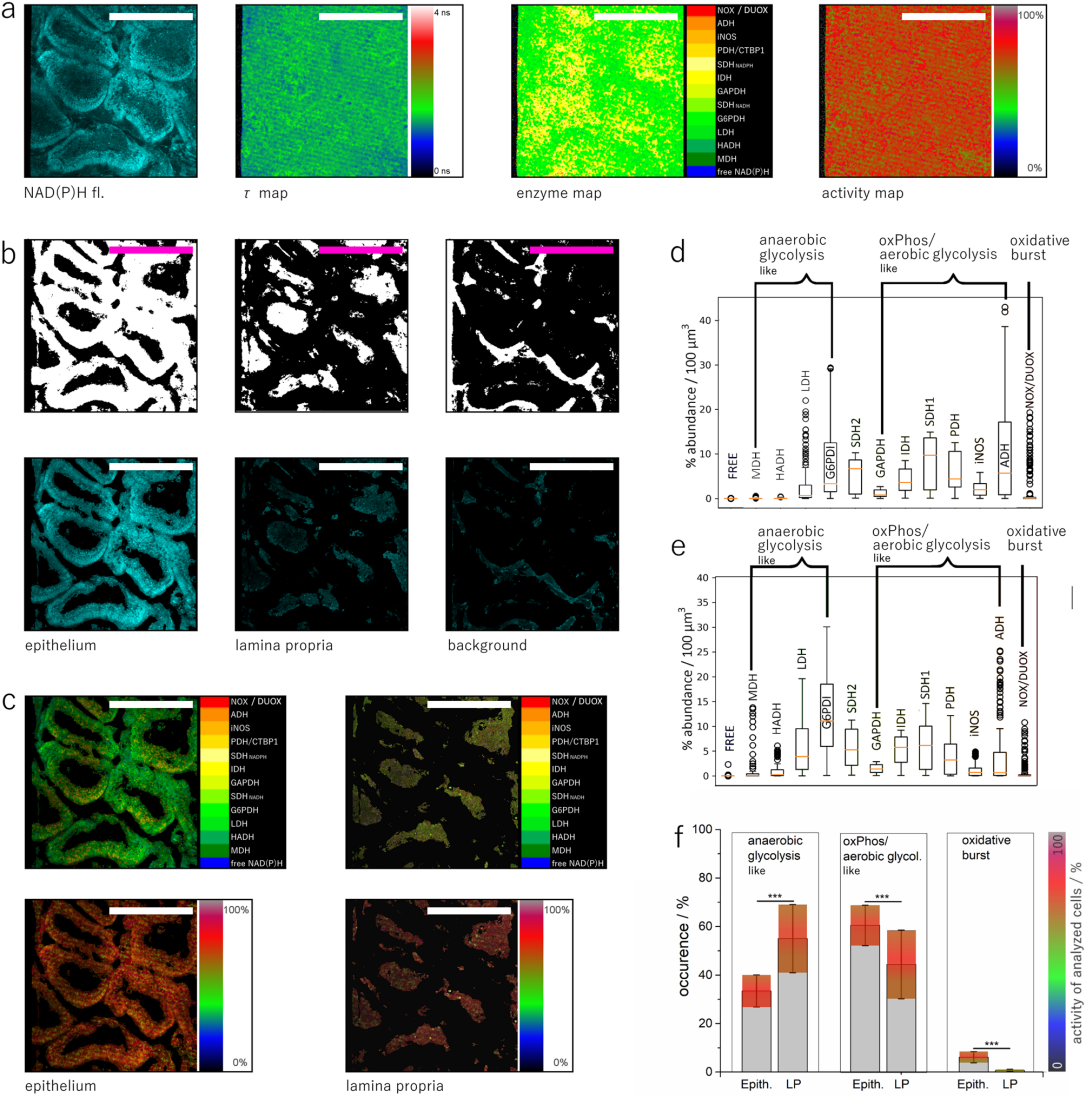
NAD(P)H fluorescence lifetime imaging of healthy explanted murine duodenum. **(a)** Representative NAD(P)H fluorescence sum image (500 × 500 × 100 µm^3^), fluorescence lifetime image, enzyme map and activity map of healthy duodenum. **(b)** Segmentation of intestinal villi in epithelium and lamina propria using the same algorithm as described in Fig. 1. **(c)** Masked enzyme and activity maps for epithelium and lamina propria. **(d)** Frequency graph of preferential enzymatic activity in epithelium (data from n = 6 mice). **(e)** Frequency graph of enzymatic activity in lamina propria (data from n = 7 mice). Scale bar = 250 µm. **(f)** Pixel frequencies of anaerobic glycolysis-like, oxidative phosphorylation (oxPhos)/aerobic glycolysis-like pathways and oxidative burst, all given in percentage, for epithelial cells and lamina propria encompassing all mice in **(d,e)**. The superimposed false color bar for each column indicates the corresponding general metabolic activity per pixel, in percent. Statistical analysis in **(f)** was performed using ANOVA test (ns p > 0.05, *p < 0.05, **p < 0.01, ***p < 0.001). Figure generated with Paint.NET, https://www.getpaint.net/download.html.

By performing NAD(P)H-FLIM in healthy, explanted duodenum (Fig. 2a) of C57/Bl6 mice, in RPMI medium, at 37 °C, we assessed the metabolic activity of intestinal epithelial cells (EC) and of cells within the lamina propria (LP), respectively, as segmented from 3D NAD(P)H fluorescence images using the previously described pixel classification algorithm (Fig. 2b,c).

Imaging in explanted live duodenum tissue led to a general metabolic activity of the EC of 78.4 ± 5.5% (n = 6 mice, field-of-view 500 × 500 µm^3^ over 50 to 110 µm depth; Suppl. Fig. 2). In some mice, the cells in lamina propria show a slightly lower metabolic activity as compared to EC, in most mice the activity levels were comparable for lamina propria and epithelium, resulting into an activity value of 76.3 ± 5.2% in LP (p = 0.5139). The variability of metabolic activity was slightly higher in lamina propria than in the epithelium, i.e. standard deviation of activity levels per mouse were 8.1% in LP as compared to 6.3% in EC. These results resemble our findings regarding metabolic activity in villi of freshly explanted duodenum as well as of duodenum in anesthetized *CX*_*3*_*CR1:GFP* mice, both acquired using NAD(P)H-FLIM^15^ (Suppl. Fig. 3).

The enzymatic activity profile in the explanted healthy duodenum shows a preference towards oxidative phosphorylation/anaerobic glycolysis-like pathways in epithelium, with local increased activation of NADPH oxidases (Fig. 2d,f). As the first line of cellular defense in the intestine, EC show a high expression of NOX4 and NOX2^15^. Both NOX2 and NOX4 activation can lead to oxidative burst, i.e. massive extracellular reactive oxygen species (ROS) generation needed in the defense against pathogens. In addition, NOX4 activation is associated with intercellular communication among intestinal epithelial cells^55^, in support of host defense. In contrast, the cells in LP balance metabolic pathways resembling both features of anaerobic glycolysis and oxidative phosphorylation/aerobic glycolysis for energy production, with a slight preference towards anaerobic glycolysis-like pathways. The LP cells show only few isolated spots of NADPH oxidase(s) activation, presumably NOX2 in phagocytes, significantly lower than the NOX activation observed in epithelium (Fig. 2e,f). These data are also in good agreement with the findings of analogous intravital experiments^12^.

### Changes in metabolic activity in the intestinal villi during *H. polygyrus* infection

**Figure 3:**
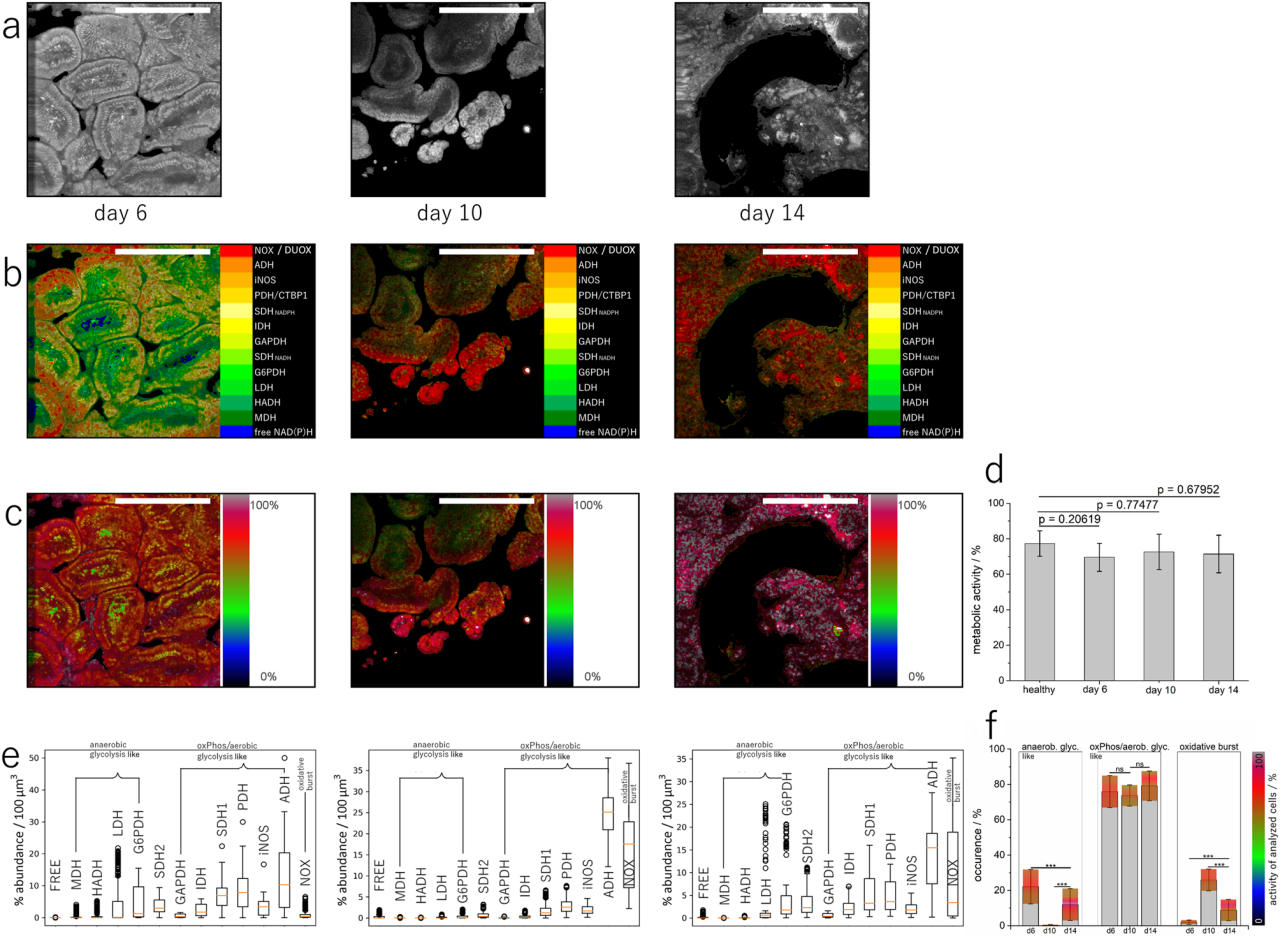
NAD(P)H fluorescence lifetime imaging of murine host duodenum from mice infected with *H. polygyrus*. **(a)** Representative NAD(P)H fluorescence sum images (500 × 500 × 100 µm^3^) of host tissue (villi) at day 6, 10 and 14 after infection (masks generated by segmentation). Corresponding enzyme maps **(b)** and metabolic maps **(c)** of the NAD(P)H fluorescence sum images in **(a)**. Scale bar for all images = 250 µm. **(d)** General metabolic activity of infected mice as compared to healthy mice (n = 2 mice for day 6; n = 3 mice for day 10; n = 4 mice for day 14 and n = 7 healthy mice—Fig. 2). **(e)** Frequency graphs for the three time points during infection encompassing data from the same infected mice as in **(d)**. **(f)** Pixel frequencies of anaerobic glycolysis-like pathways, oxidative phosphorylation (oxPhos)/aerobic glycolysis-like pathways and oxidative burst, all given in percentage, for villi tissue (including epithelium and lamina propria, without further segmentation) encompassing all mice in **(e)** and Fig. 2d,e. The superimposed false color bar for each column indicates the corresponding general metabolic activity per pixel, in percent. Statistical analysis in **(d,f)** was performed using ANOVA test with Bonferroni multi-column post-test (ns p > 0.05, *p < 0.05, **p < 0.01, ***p < 0.001). Figure generated with Paint.NET, https://www.getpaint.net/download.html.

NAD(P)H fluorescence images of duodenum tissue from mice infected with *H. polygyrus* revealed a disorganization of the typical villi architecture in the vicinity of nematodes (Fig. 3a, Suppl. Fig. 4). Consequently, segmenting epithelium and lamina propria, as described for the healthy duodenum, was not always possible in the infected gut and we refer in all following data to intestinal tissue, without differentiating between epithelium and lamina propria.

When compared to healthy duodenum (77.3 ± 5.2%), NAD(P)H-FLIM analysis of infected duodenum tissue, 6 to 14 days post infection (acute infection), revealed a similarly high metabolic activity (74.0 ± 7.6%, n = 9 mice, Fig. 3c,d), with high heterogeneity within the villi (s.d. 9.6%). A preference towards metabolic pathways resembling features of oxidative phosphorylation/aerobic glycolysis was observed in all mice (Fig. 3b,e,f). In contrast, at an earlier time point of infection, i.e. at day 6 after infection, where most parasites still reside in the submucosa, the typical villus morphology is preserved. At this time point during infection, in some areas in one mouse, a preference towards anaerobic glycolysis-like pathways was observed (Fig. 3b, left image), however, in most duodenal villi, oxidative phosphorylation/aerobic glycolysis-like pathways prevailed (n = 2 mice, Fig. 3e,f).

In line with the expected time course of immunomodulation caused by nematodes in the host^6,8,11,56–59^, NADPH oxidases activation in the villi is rather low, i.e. comparable to healthy tissue, at day 6 after infection but increases dramatically at day 10 and 14 after infection, i.e. during the acute phase, in the vicinity of parasites (Fig. 3e,f).

### NAD(P)H metabolic and enzymatic activity of live *H. polygyrus* in the gut

**Figure 4:**
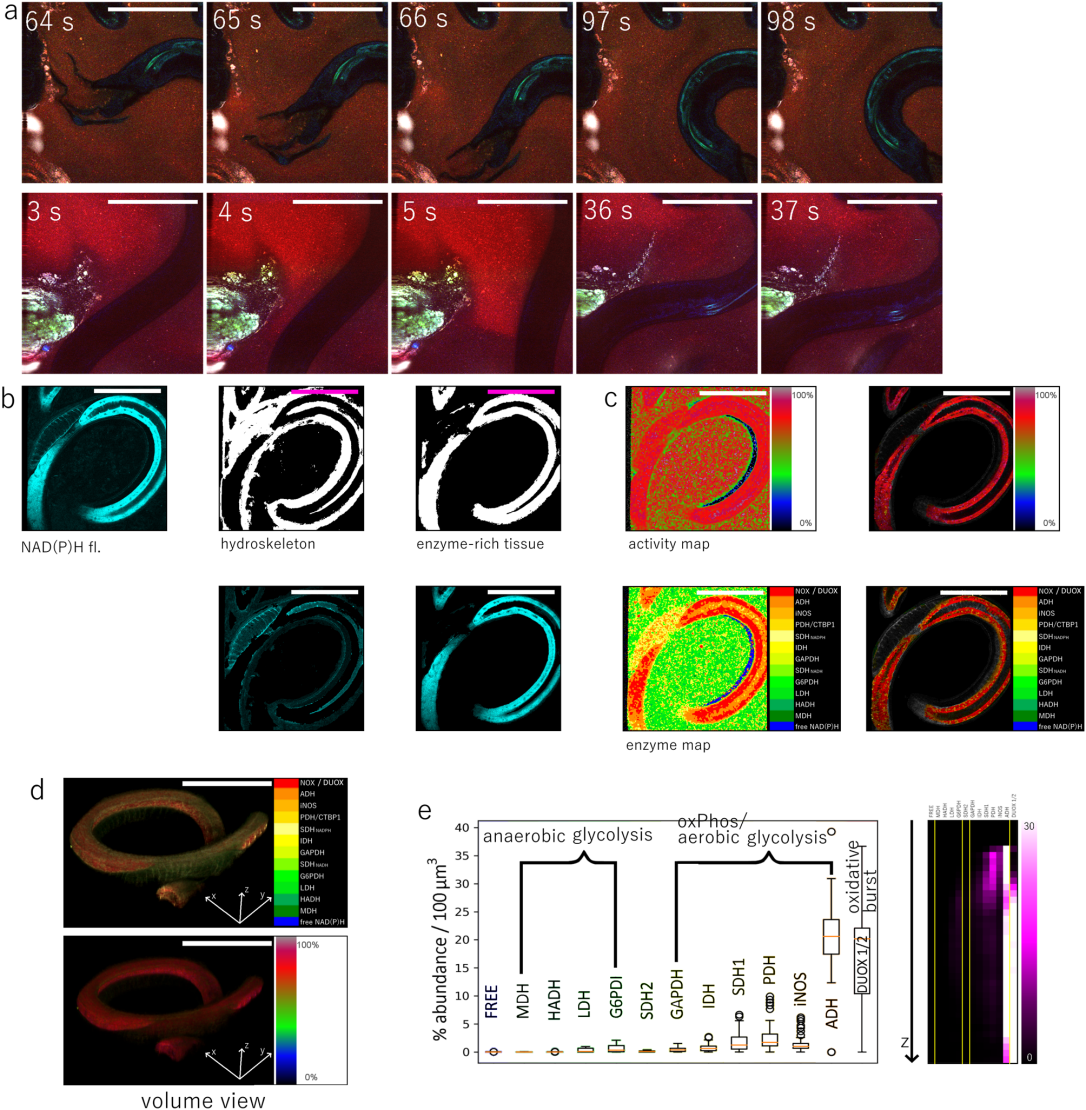
NAD(P)H fluorescence lifetime imaging of *H. polygyrus* in intestinal environment. **(a)** Snapshots (500 × 500 µm images) of *H. polygyrus* movement in the murine intestinal lumen. Endogenous fluorescence of parasite and host villi (green and blue), BODIPY1 fluorescence in mucus (red). λ_exc_ = 920 nm. **(b)** Representative NAD(P)H fluorescence sum image (500 × 500 × 100 µm^3^) and segmentation of the worm compartments, differentiating between hydro-skeleton (low NAD(P)H fluorescence) and worm tissue (NAD(P)H-rich areas). **(c)** Enzyme and activity maps of the entire NAD(P)H signal (right panel) and masked enzyme and activity maps of the NAD(P)H-rich worm tissue (left panel). **(d)** 3D reconstructions of the enzyme and activity maps shown in the left panel in **(c)**. **(e)** Frequency graph of enzymatic activity in the entire parasite and depth-dependent frequency representation of enzymatic activity. Scale bar for all images = 250 µm. Figure generated with Paint.NET, https://www.getpaint.net/download.html.

Adult *H. polygyrus* nematodes residing in the intestinal lumen display active motility of loose ends of their body, i.e. the ends not being coiled around villi (Fig. 4a, Suppl. Movie 1, 2). This active, undulatory motion through the intestinal mucus (labelled by the lipophilic dye BODIPY1) indicates the vitality of the worms and probably is key for their locomotion upwards in the small intestine and their survival in the duodenal luminal niche. In order to understand the energetics governing their motility behavior, we aimed to investigate NAD(P)H metabolism of nematodes by NAD(P)H-FLIM.

Using the same pixel classification approach as previously described, the nematode hydrostatic skeleton and its cellular, NAD(P)H-rich tissue were segmented from the NAD(P)H intensity images of *H. polygyrus* in the murine duodenum (Fig. 4b). The resulting mask was used to generate metabolic and enzyme 2D and 3D maps of the worms (Fig. 4c,d) and to calculate the relative preferential activation of the different metabolic pathways (Fig. 4e). Interestingly, several worms showed extensive NADPH oxidase activation, presumably DUOX2 activation, as the only member of the NADPH oxidases family expressed in *H. polygyrus*^53^. The activation of DUOX2 appeared to be particularly high in the digestive system of the nematode and may indicate parasite defense against the host.

### Metabolic and specific enzymatic activity of *H. polygyrus* depends on the specific phase during infection

**Figure 5:**
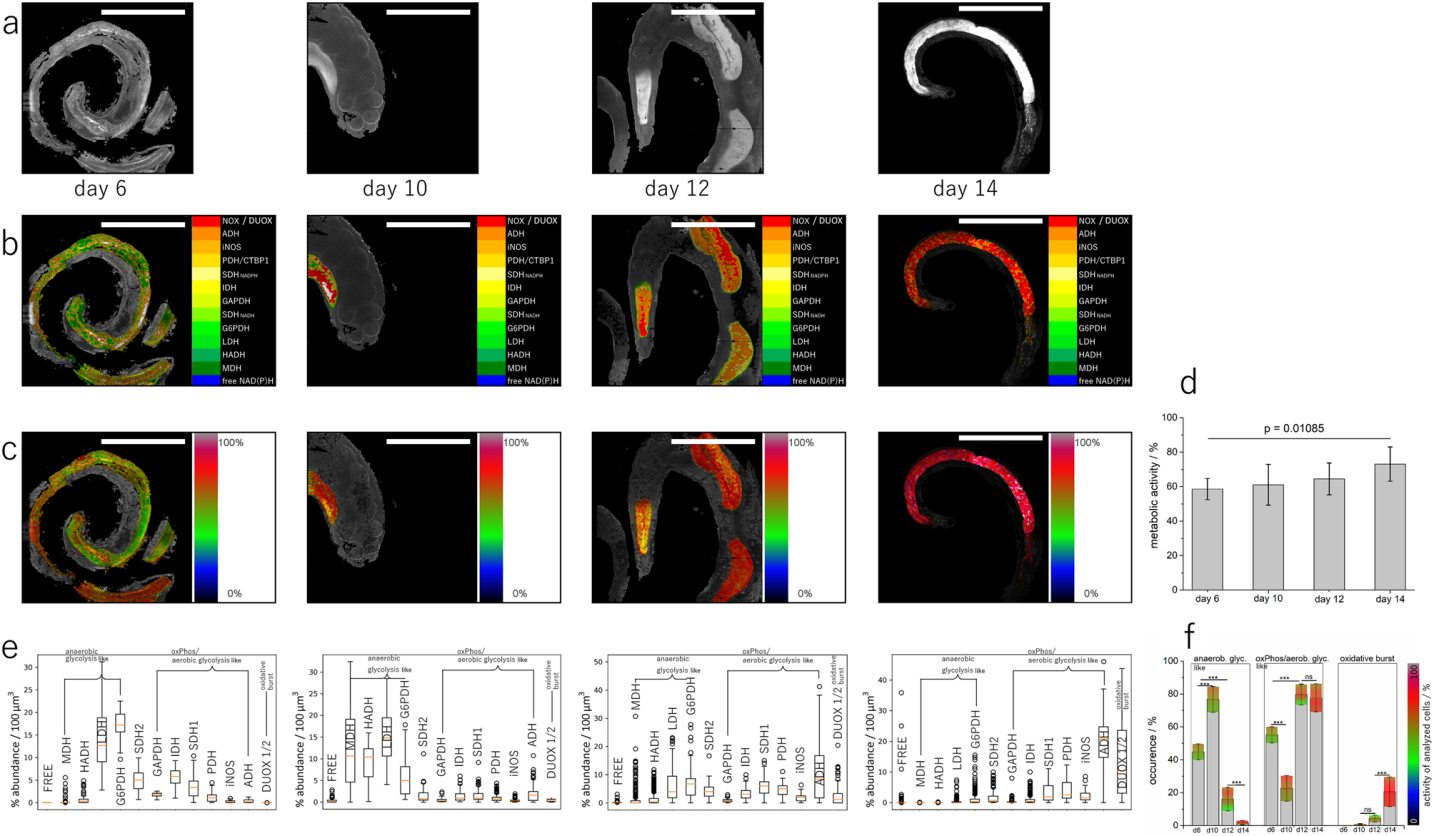
NAD(P)H metabolism of *H. polygyrus* over the course of infection. **(a)** Representative masked NAD(P)H fluorescence sum images (500 × 500 × 100 µm^3^) of NAD(P)H-rich parasite compartments at day 6, 10, 12 and 14 after infection and corresponding enzyme activity maps **(b)** and activity maps **(c)**. Scale bar = 250 µm. **(d)** Metabolic activity graph in parasites, at the same time points (n = 2 worms, day 6; n = 5 worms for day 10; n = 4 worms, day 12; n = 5 worms, day 14). **(e)** Corresponding frequency graphs of enzymatic activity for the same parasites as in **(d)**. **(f)** Pixel frequencies of anaerobic glycolysis-like pathways, oxidative phosphorylation (oxPhos)/aerobic glycolysis-like pathways and oxidative burst, all given in percentage, encompassing all nematodes in **(d)**. The superimposed false color bar for each column indicates the corresponding general metabolic activity per pixel, in percent. Statistical analysis in **(f)** was performed using ANOVA test with Bonferroni multi-column post-test (ns p > 0.05, *p < 0.05, **p < 0.01, ***p < 0.001). Figure generated with Paint.NET, https://www.getpaint.net/download.html.

In order to understand if the translocation from the larval development in the submucosa to an adult worm in the gut lumen is associated with a specific shift between different metabolic pathways, we tracked the changes in NAD(P)H metabolism of *H. polygyrus* parasites between day 6 and day 14 after infection, using NAD(P)H-FLIM (Fig. 5a–c).

As compared to the host intestinal tissue, nematodes show a lower metabolic activity (64.3 ± 6.3%, n = 16 worms from 14 mice, between day 6 and 14 after infection), with a similarly high heterogeneity per worm of 9.3%. Interestingly, the general metabolic activity in worms slightly increases from day 6 to day 14 after infection, i.e. day 6 metabolic activity 58.6 ± 2.2% (2 worms), heterogeneity per single worm 6.1%, day 10 61.1 ± 9.9% and 11.8% (5 worms), day 12 64.5 ± 5.8% (4 worms) and day 14 73.1 ± 5.9% (5 worms) (Fig. 5d).

At day 6 after infection, *H. polygyrus* parasites are located in the intestinal submucosa. At this stage, they generate energy using metabolic pathways resembling features of both anaerobic glycolysis and oxidative phosphorylation/aerobic glycolysis and show very low levels of oxidative burst (DUOX2 activation). 10 days after infection, the parasites start to emerge from the submucosa into the lumen. This process is associated with a metabolic shift towards anaerobic glycolysis-like pathways, at still low DUOX2 activation. Between day 12 and day 14 after infection, the adult parasites shift their metabolism towards oxidative phosphorylation/aerobic glycolysis-like pathways and show increased levels of DUOX2 activation (oxidative burst), indicating defense mechanisms, possibly against the host (Fig. 5e,f).

## Discussion

Understanding physiological and pathological phenomena require the spatio-temporal analysis of cell phenotypes and functions in their genuine environment, the living tissue or even living organism. Due to reduced scattering of the excitation wavelengths and tissue-friendly confinement of excitation around the focal point of the objective lens, two-photon fluorescence laser-scanning microscopy^28^, and more recently, three-photon fluorescence laser-scanning microscopy^60^ have proven to be the most versatile tools fulfilling this requirement. In the past decades, these technologies brought new insights about cellular dynamics and communication both in health and in disease, particularly in neurosciences^61–63^, immunology^64–68^ and oncology^69–71^.

Additionally, extending two-photon microscopy with fluorescence lifetime imaging (FLIM)^41^ allowed access to quantitative information regarding cellular and tissue functions in vivo. For instance, FLIM has been used in combination with FRET-based calcium constructs expressed in transgenic mice to monitor absolute calcium levels in a cell-specific manner^72,73^. In order to monitor cellular metabolism, being the cornerstone of cellular and tissue function, in vivo FLIM of the ubiquitous metabolic co-enzymes nicotinamide adenine dinucleotide (NADH) and nicotinamide adenine dinucleotide phosphate (NADPH) has been performed. This shows both the general metabolic activity of cells as well as information on specific enzymes, which may preferentially bind to NAD(P)H, i.e. may participate in biochemical reactions. In this way, the NAD(P)H-dependent metabolism could be cross-linked to cellular vitality/dormancy^49^ and cellular differentiation, under physiologic conditions^32,74^ and in tumors^19^. Additionally, it could be related to (regular or aberrant) defense mechanisms in various tissues^13,14,51^. In order to extend the access to information contained in NAD(P)H-FLIM data, we previously proposed a systematic phasor-approach based evaluation frame-work of these data^24^, which retrieves the ratio of free and enzyme-bound NAD(P)H and the preferential binding of the coenzymes to the most abundant NAD(P)H-dependent enzymes across species.

Whereas, up to now, several studies employed in vivo NAD(P)H-FLIM to investigate metabolism in mammals, especially in mice, there are any studies concerning the NAD(P)H-dependent metabolism of intestinal pathogens such as nematodes and its cross-link to the metabolism of the host tissues.

By using NAD(P)H-FLIM upon two-photon excitation at 760 nm, we studied the metabolism of explanted intestinal duodenal tissue and, in parallel, of the parasitic worm *Heligmosomoides polygyrus,* in wild type C57/Bl6 mice. We found that the metabolic activity patterns of the intestinal villi, indicative for metabolic fitness, in explanted duodenum are comparable to intestinal tissue imaged under intravital conditions^15^. Besides, a similar pattern of NADPH oxidases activation, i.e. higher in epithelial cells than in cells of the lamina propria, was detected both in explanted duodenum, as presented here, and in the small intestine of living mice^12^. In this way, we validated the experimental model for analyzing the cross-link between host and parasite, along the metabolic axis.

With regard to the metabolic activity of the host tissue during the presence of nematodes in the intestinal lumen, during the acute phase of the infection, we revealed a strong defense reaction by the host, in the direct vicinity of parasites. This effect was not observed in the villi before adult worms reemerge from the submucosa, where invading stage 3 larvae develop, via an L4 larval stage, into adults. This defense reaction in the intestinal lumen presumably indicated by a strong activation of NADPH oxidases in epithelium and in the lamina propria was associated with a disintegration of the typical tissue architecture of the villi, in direct nematode vicinity. This finding is in line with a strong anti-nematode host immune response associated with GATA-3 + Th2 cells and GATA-3 + T-bet + Th2/1 hybrid cells, production of the respective cytokines (IL-4, IL-5, IL-13, IFN-γ) as well as a prominent eosinophilia at the side of infection^1,75^. Thus, during the acute phase of infection, the strong activation of NADPH oxidase and tissue disintegration coheres with a prominent anti-pathogen immune response. A silencing of the host defense, in line with the shift towards an immunoregulatory response suppressing the Th2 activity^4^, is expected during chronic infection—a stage not monitored in this study.

The metabolic activity of the parasite paralleled their motility level and, possibly, their differentiation stage. *H. polygyrus* parasites balanced energetic metabolic pathways resembling features of effective oxPhos/aerobic glycolysis (associated with low motility) and of energy-demanding anaerobic glycolysis (associated with either maturation or active movement), when residing in the submucosa. Our data show that, when the nematodes emerge from the submucosa into the intestinal lumen, they preferentially shift their metabolism towards anaerobic glycolysis-like pathways for rapid (but ineffective) energy production. After arriving in their persistence niche in the duodenum, they shift back to effective energy production, i.e. mainly towards oxPhos/aerobic glycolysis-like pathways, presumably in order to effectively budget the nutrient and oxygen resources, which parasite and host compete for.

DUOX2—the only member of the NADPH oxidases family expressed in *H. polygyrus*^53^—is highly activated in the gastrointestinal system and partially in the cuticle of parasites residing in the duodenal persistence niche. In contrast, parasites in the submucosa do not show DUOX2 activation. Presumably, due to the molting process of the parasite from larval stage 3 via larval stage 4 into adults which is associated with stage-specific parasite antigens. During this process, there is probably no need to invest additional energy to defend themselves via oxidative burst. DUOX2 activation in parasites increases during the course of infection up to day 14, when emerging into the intestinal lumen. The presence of DUOX1/2 was previously reported in cuticle and intestine of *C. elegans*^76^, a soil nematode, in line with our observations regarding the location of activated DUOX2 in *H. polygyrus*.

In conclusion, the here presented live tissue NAD(P)H-FLIM approach to study metabolism of intestinal nematodes in a genuine tissue context provides us with the unique opportunity to decipher a new dimension of the host-parasite crosstalk. In future, this will be relevant for multiple issues such as the investigation of detrimental effects of the long-term chronic phase of the parasitic infection, a more efficient exploitation of nematode infections in the immunomodulatory treatment of autoimmune diseases, or for studying the interaction of the intestinal nematodes with gut microbes as well as concurrent co-infecting gut pathogens.

## Materials and methods

### Two-photon laser-scanning microscope setup for fluorescence lifetime imaging of NAD(P)H

Two-photon fluorescence lifetime imaging experiments were performed using a specialized laser-scanning microscope based on a commercial scan head (TriMScope II, LaVision Biotec—a Miltenyi company, Bielefeld, Germany). A near-infrared laser (Ti:Sa, Chameleon Ultra II, Coherent, Duisburg, Germany) tuned at 760 nm, repetition rate 80 MHz, and pulse width 140 fs was used as excitation source for NAD(P)H. The linearly polarized Ti:Sa beam was scanned over the sample by galvanometric mirrors. A water-immersion objective lens (20×, NA 1.05, Apochromat, Olympus, Hamburg Germany) was used to focus the laser beam into the explanted duodenum samples. The laser power was controlled by a combination of λ/2 wave-plates and polarizers. The ultrashort pulses of the laser were compressed using an external compressor. NADH and NADPH fluorescence was collected in the backward direction using a dichroic mirror (775, Chroma, Marlborough, MA, USA), passed through an interference filter (466 ± 30 nm) and was detected by a GaAsP PMT (Hamamatsu, Herrsching, Germany) connected to the previously described TCSPC electronics^31^ (LaVision Biotec—a Miltenyi company, Bielefeld, Germany). The fluorescence of the ubiquitous coenzymes NAD(P)H was detected at 466 ± 30 nm after two-photon excitation at 760 nm. The TCSPC data were collected at a time resolution of 55 ps, over at least 9 ns and with a Gaussian-shaped instrument response function of 250 ps FWHM. In all imaging experiments, we used an average maximum laser power of 30 mW to avoid sample photodamage. The acquisition time for an image with a field-of-view of 500 µm × 500 µm and a digital resolution of 505 × 505 pixel was 800 ms. The lipophilic dye BODIPY1 used to label the intestinal mucus was excited at 920 nm and its fluorescence was detected by a GaAsP PMT (Hamamatsu, Herrsching, Germany) through an interference filter (594 ± 20 nm).

### Phasor analysis of time-domain FLIM data

Fluorescence lifetime data were analyzed as previously described^24^. The phasor approach transforms the time-domain data (the fluorescence decay), into a virtual, normalized phase domain by calculating the discrete Fourier transformation numerically (modulations frequency = 80 MHz). The transformation leads to a complex number, the real and imaginary parts of which give the coordinates of the vector in the phase domain (“phasor”). That vector originated in (0|0) and points towards the half circle (centrum at (0.5|0), radius = 0.5), due to the exponential nature of the original time domain data. Because of the value normalization, the real part of the phasor reaches from 0 to 1, and those of the imaginary part from 0 to 0.5. In this way, short fluorescence lifetimes of homogeneous fluorophores (mono-exponential decay) are located on the half circle at large real values, whereas with increasing lifetime, the real value decreases.

As previously described^24^, the general metabolic activity (displayed in the activity map) is geometrically calculated as the distance from each experimental data point in the phasor plot to the intersection of the segment connecting the data point and the predefined position of free NAD(P)H lifetime with the half circle. Thus, the activity map defines the ratio between free and enzyme-bound NAD(P)H, independent of the enzyme to which the coenzymes bind to. Since this distance relates to the total length of the segment, from the free NAD(P)H position to the second intersection of the line with the half circle (representing fully enzyme-bound NAD(P)H), the activity is expressed in percentage: 0% only free NAD(P)H, 100% only enzyme-bound NAD(P)H.

Moreover, the enzymatic activity (enzyme map) relies in each pixel on the probability that NAD(P)H is bound to each enzyme among the most abundant enzymes as revealed by RNASeq of both mammal and *H. polygyrus* tissues. This probability is calculated based on a similarity approach, comparing the angles between the predefined segments connecting the free NAD(P)H position and the specific enzyme-bound NAD(P)H positions with the line connecting each experimental data point with the free NAD(P)H position. Thus, the sum of all probabilities in each pixel equals 100%. For each pixel in the enzyme map, we determined the enzyme of maximum probability for binding to NAD(P)H, i.e. even if only slightly higher as compared to enzymes with similar binding sites for the coenzymes, and displayed this in a color-coded manner. We account for similar binding sites for the coenzymes, i.e. similar directions of the phase vectors and similar NAD(P)H fluorescence lifetimes, by representing them in similar colors and by grouping them together, to estimate possible metabolic pathways.

### Mice and infection model

All mice used were C57Bl/6 mice. 200 stage 3 (L3) *H. polygyrus* larvae were given orally to female 10–12 weeks old mice, as previously described^4^. Imaging experiments were performed during the acute phase of the infection between day 6 and day 14 after infection.

All animal experiments were performed in accordance with the National Animal Protection Guidelines and approved by the Berlin Ethics Committee for the Protection of Animals (G0176/16).

All animal experiments were performed in accordance with the ARRIVE guidelines. The present study compares the duodenum of healthy C57/Bl6 mice (control group) with those of mice infected with the intestinal nematode *H. polygyrus*, during the acute phase of infection, day 6 to day 14 after infection. In the group of infected mice, we performed a further stratification of the results referring to the time point after infection (at day 6, day 10 and day 14). n refers to number of animals, with 20 to 50 tissue layers from a 1 cm piece of duodenum in each animal. In the control group, we included 7 mice and in the group of infected mice we included 9 mice. We omitted a calculation of sample size due to the exploratory character of the present study. Due to the same reason, we performed no randomization and no blinding.

### Preparation of the freshly explanted duodenum samples for imaging

Mice were sacrificed by cervical dislocation. The small intestine was freshly explanted, the duodenum was cut of the rest of the small intestine, and placed on ice. After opening up the duodenum tube to expose the lumen with adult parasites, the duodenum was fixed on a Petri dish using tissue glue and immersed in RPMI medium containing 10% FCS. The Petri dish was either directly placed on the microscope table for two-photon imaging or kept on ice at 4 °C and after a maximum duration of 3 h placed on the microscope table for subsequent imaging at the time points indicated in Suppl. Fig. 2. A temperature of 37 °C was maintained during the entire imaging experiment using a heating foil. In some experiments, the intestinal mucus was labelled by BODIPY1 (Thermo Fisher, Bremen, Germany).

### Data analysis

By summing up the intensity values of the acquired time-resolved NAD(P)H fluorescence images, the phasor-analysis Python routine provides an intensity image for each depth in tissue, containing only spatial information. The resulting z-stacks are typically 505 × 505 × ≈ 80 voxel. We first individually normalized the contrast and brightness of all depth slices within a z-stack using a dedicated Fiji-macro. For segmentation of different tissue areas, we used the ‘Pixel-Classification’ function in ILASTIK. Thereby, we created neural networks with adapted parameters for all conditions. In detail, we manually assigned the pixels of about one third of the slices in the z-stack and used these for training the neural network. The slices were evenly distributed over the entire depth of the stack. Another third of the slices were used for validation. Feature selection included intensity, edges, texture and structure with weights increasing in this order and with ALPHA values between 0.3 and 100. Training data was labeled with a maximum of three classes: (i) for healthy murine intestinal tissue: background, epithelium and lamina propria, (ii) for infected tissue: background, host intestinal tissue and worm and (iii) for segmented parasites: background, hydro-skeleton and enzyme-rich tissue. The masks generated by ILASTIK were applied to the enzyme maps, activity maps and intensity projections. Subsequently, we weighted the in the preprocess Gaussian-filter-smoothed enzyme and activity maps with the segmented intensity images using a Fiji macro and assigned a LUT according to the pixel values (enzyme-map 1–13, activity-map 0–100%). The sum of each enzyme for each masked slice of a measurement was counted and stored in a text file. From this, we generated the z-abundance plots with (enzymes × stack-depth × abundance as pixel intensity). Correspondingly, the mean value and the standard deviation of the activity of each slice of the measurement was calculated. Using Matplotlib and numPy in a Python script, we then created box-plots for the enzyme-frequency plots from the text-stored-values over the depth for each measurement or measurement series.

Using the enzyme activity abundance information in each depth of a z-stack—either for the murine tissue or for the enzyme-rich tissue of the nematodes, we calculated the relative occurrence of metabolic pathways resembling features of anaerobic glycolysis, i.e. the sum of the abundance of the enzymes grouped around LDH, as compared to that of oxPhos/aerobic glycolysis, i.e. the sum of the abundance of the enzymes grouped around PDH, standing for mitochondrial metabolic activity, and to the oxidative burst, i.e. abundance of NOX and DUOX enzymes.

For statistical analysis, we used ANOVA tests (ns p > 0.05, *p < 0.05, **p < 0.01, ***p < 0.001). In case of multi-column comparison, we used the Bonferroni test. All data in the manuscript are presented as mean standard ± deviation.

### Ethical approval

All animal experiments were performed in accordance with the National Animal Protection Guidelines and approved by the Berlin Ethics Committee for the Protection of Animals (G0176/16) as part of the Landesamt fuer Gesundheit und Soziales (LaGeSo), Berlin (the State Office for Health and Social Affairs).

## Supplementary Information

Supplementary Legends.

Supplementary Figure 1.

Supplementary Figure 2.

Supplementary Figure 3.

Supplementary Figure 4.

Supplementary Figure 5.

Supplementary Movie 1.

Supplementary Movie 2.

## Data Availability

The datasets used and analyzed during the current study are available from the corresponding author on reasonable request.
