# Supplementary material for: NAD(P)H fluorescence lifetime imaging of live intestinal nematodes reveals metabolic crosstalk between parasite and host
Source: Sci Rep. 2022 May 4;12:7264. doi: 10.1038/s41598-022-10705-y (PMC9068778; doi:10.1038/s41598-022-10705-y)
Supplement: Supplementary file 1 — Supplementary Legends. [file 41598_2022_10705_MOESM1_ESM.docx]

**Supplemental information**

**NAD(P)H fluorescence lifetime imaging of live intestinal nematodes reveals metabolic crosstalk between parasite and host**

Wjatscheslaw Liublin^1,2^, Sebastian Rausch^3^, Ruth Leben^1,2^, Randall L. Lindquist^4^, Alexander Fiedler^1,2^, Juliane Liebeskind^5,6^, Ingeborg E. Beckers^7^, Anja E. Hauser^5,6^, Susanne Hartmann^3*^, Raluca A. Niesner^1,2*^

^1^Biophysical Analytics, Deutsches Rheuma-Forschungszentrum, Berlin, a Leibniz Institute, Berlin, Germany

^2^Dynamic and functional *in vivo* imaging, Institute for Veterinary Physiology, Department of Veterinary Medicine, Freie Universität, Berlin, Germany

^3^Institute of Immunology, Department of Veterinary Medicine, Freie Universität Berlin, Germany

^4^Department of Nuclear Medicine, Charité – Universitätsmedizin, Berlin, corporate member of Freie and Humboldt University, Berlin, Germany

^5^Laboratory for Immune Dynamics, Deutsches Rheuma-Forschungszentrum, Berlin, a Leibniz Institute, Berlin, Germany

^6^Intravital Microscopy and Immune Dynamics, Department of Rheumatology and Clinical Immunology, Charité – Universitätsmedizin, Berlin, corporate member of Freie and Humboldt University, Berlin, Germany

^7^Berliner Hochschule für Technik, School for Applied Sciences, Berlin, Germany

*Equally contributing senior authors.

Correspondence: Prof. Dr. Raluca A. Niesner, [raluca.niesner@fu-berlin.de](mailto:raluca.niesner@fu-berlin.de)

**Supplemental figure captions**

**Supplemental Figure 1. Calculation of the SNR for the NAD(P)H-FLIM data. (a)** Left to right: representative fluorescence sum image of villi in the duodenum of a healthy Bl6 mouse, mask of epithelial tissue and lamina propria, segmented as presented in the manuscript, depth = 80µm, scale bar = 250µm. **(b)** Mean SNR of the entire image, the masked epithelium and lamina propria, respectively. We first defined the segmented ROIs as signal and background. Subsequently, the mean SNR values were calculated as the difference of the mean over the signal area (µSIG) and the mean over the background area (µBG), divided by the standard deviation of the background histogram σBG, for each image. **(c)** Mean SNR values for all datasets, for LP=lamina propria, EP=epithelium, TIS=entire tissue of the host, and ERT=NAD(P)H-enzyme-rich tissue of the worm, i.e. high NAD(P)H fluorescence signal. The threshold of SNR=5 as shown to be critical for accurate data evaluation^21^ was surpassed in all datasets. Figure generated with Paint.NET, <https://www.getpaint.net/download.html>.

**Supplemental Figure 2 Preparation routine and experimental pipeline (a)** Timescale corresponding to the preparations and actions shown in **(b)** Sacrificing mouse at ~ t-20 min, opening, localizing and harvesting the duodenum, cutting off ~ 4 pieces of (5-10) mm, opening the tube with blunt scissors and gluing it luminal side upwards with tissue glue on a petri dish. The samples are then covered with medium, one is directly placed on an incubator plate, heated up to 37°C and under the microscope for measurements while the others are placed on ice. After measuring (typically 36 min including searching for an appropriate ROI (~ 10-15 min) and measuring time itself (~ 20 – 25 min)) samples are switched. The next sample is heated to 37°C, while an ROI is found and the microscope is adjusted for the measurement. Steps are repeated until last sample is measured. Queueing times are staggered for the samples, whereas first sample had no waiting time, second ~ 36 min - until last sample (no longer than 180 min, i.e. 3 hours in total) was processed. **(c)** Measuring time per sample graphs for all datasets. Mean measuring time was 36 minutes, typically with a variation of few minutes, as shown in the graph. Figure generated with Paint.NET, <https://www.getpaint.net/download.html>.

**Supplemental Figure 3.** **NAD(P)H-dependent metabolic activity and tissue architecture of the duodenum under *in vivo* conditions as compared to freshly explanted duodenum tissue and to duodenum tissue kept on ice for 3 hours (a)** Exemplary activity map of duodenum of a healthy Bl6 mouse, imaged *in vivo*. Scale bar = 100 µm and segmentations of epithelium and lamina propria respectively. **(b)** Mean activity of epithelium and lamina propria of n = 5 mice imaged *in vivo*. **(c)** Activity maps of the same ROI of one freshly explanted duodenum sample at 5 min and 68 min post sacrifice and segmentation of epithelium and lamina propria. The sample was imaged over the time course of 110 min in 20 min steps and for a depth of 150 µm while remaining under the microscope under the specified experimental conditions (37°C). Scale bar = 250 µm **(d)** Mean activity of epithelium and lamina propria of n = 1 mouse imaged over 110 min. **(e)** Activity map of explanted duodenum after being stored 3 hours on ice, warming up and then being imaged for 30 min, comparable to samples from the experiment that had been kept on ice for the longest queueing time. **(f)** Mean activity of epithelium and lamina Propria of n = 1 mouse imaged after 3h on ice. Figure generated with Paint.NET, <https://www.getpaint.net/download.html>.

**Supplemental Figure 4. Comparing tissue structure, activity and enzyme abundance of ROIs in close and far proximity to the parasite in infected mice.**  Comparison of metabolic and enzymatic activity relying on the phasor-analyzed NAD(P)H-FLIM data of villi in nematode vicinity and distal from nematodes, at day 10 after infection in the same individual (EX2A4). **(a)** Fluorescence sum image of a ROI with worm, segmentation for tissue of the host, enzyme and activity map **(b)** Fluorescence sum image of a ROI distal from the parasite, segmentation for tissue of the host, enzyme and activity map **(c)** Enzyme abundance map for ROIs a and b. Figure generated with Paint.NET, <https://www.getpaint.net/download.html>.

**Supplemental Figure 5. 3D representations of pixel density referring to metabolic activity and metabolic pathways graphs corresponding to the 2D representations in Figures 2f, 3f and 5f.**  Pixel frequencies of anaerobic glycolysis-like, oxidative phosphorylation (oxPhos) / aerobic glycolysis-like metabolic states and oxidative burst along x axis, pixel density on y axis and metabolic activity in each pixel (in %) along the z-axis, plotted as height information. 3D representations **(a)** for healthy host intestine as in Figure 2f, **(b)** for infected host intestine, proximal to nematodes corresponding to Figure 3f and **(c)** worms as shown in Figure 5f.  Figure generated with Paint.NET, <https://www.getpaint.net/download.html>.

**Movie captions**

**Supplemental movie 1.** Movement of an adult, male *H. polygyrus* in the murine intestinal lumen. The endogenous fluorescence of the nematode (blue and green) and of intestinal mucus labeled by BODIPY1 (red) was acquired over an area of 500x500 µm², every 2 s. Scale bar = 250 µm.

**Supplemental movie 2.** Movement of an adult *H. polygyrus* in the murine intestinal lumen. The endogenous fluorescence of both nematode and host tissue (blue and green) and of the mucus labeled by BODIPY1 (red) was acquired over an area of 500x500 µm², every 2 s. Scale bar = 250 µm.
