# Supplementary figures and images for: NAD(P)H fluorescence lifetime imaging of live intestinal nematodes reveals metabolic crosstalk between parasite and host
Source: Sci Rep. 2022 May 4;12:7264. doi: 10.1038/s41598-022-10705-y (PMC9068778; doi:10.1038/s41598-022-10705-y)

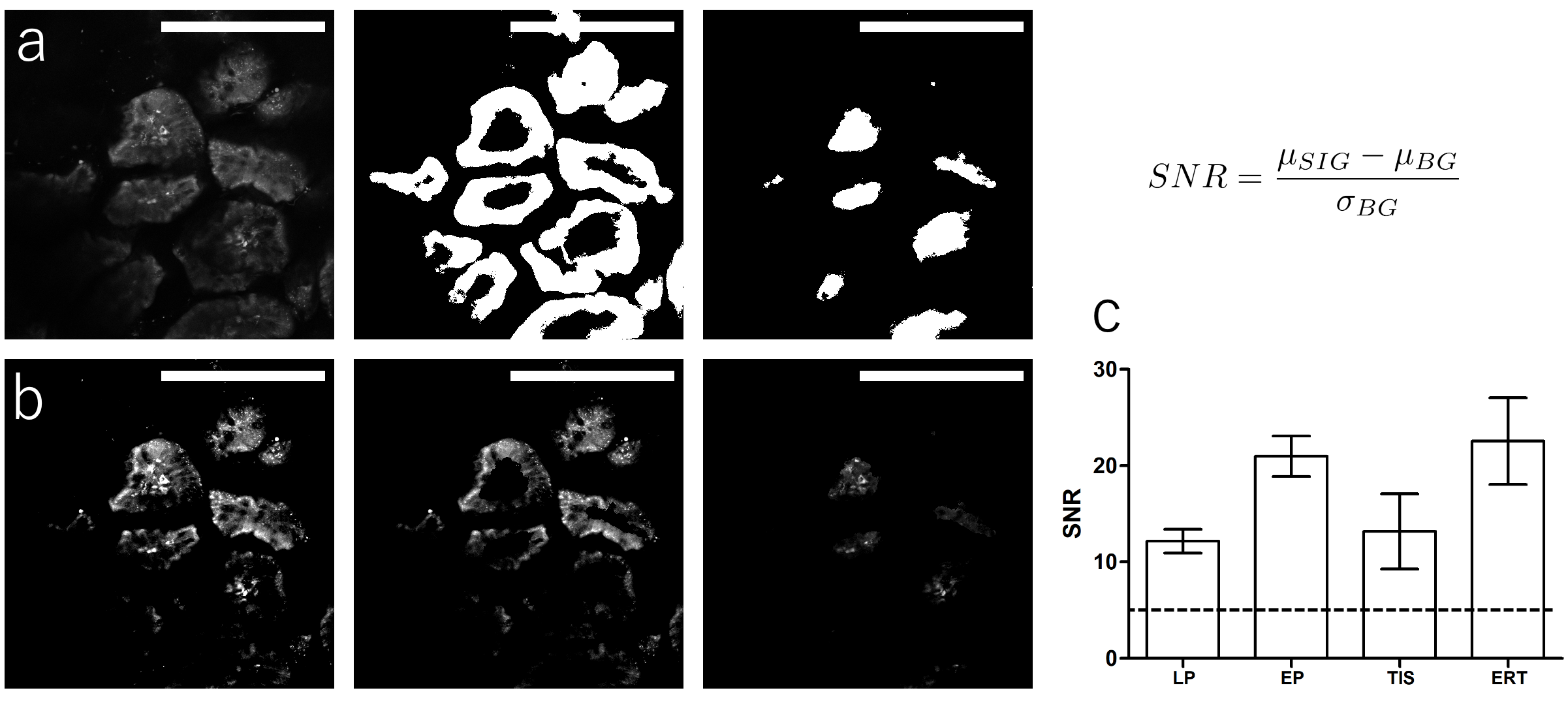

Supplement: Supplementary file 2 — Supplementary Figure 1. [file 41598_2022_10705_MOESM2_ESM.tiff]

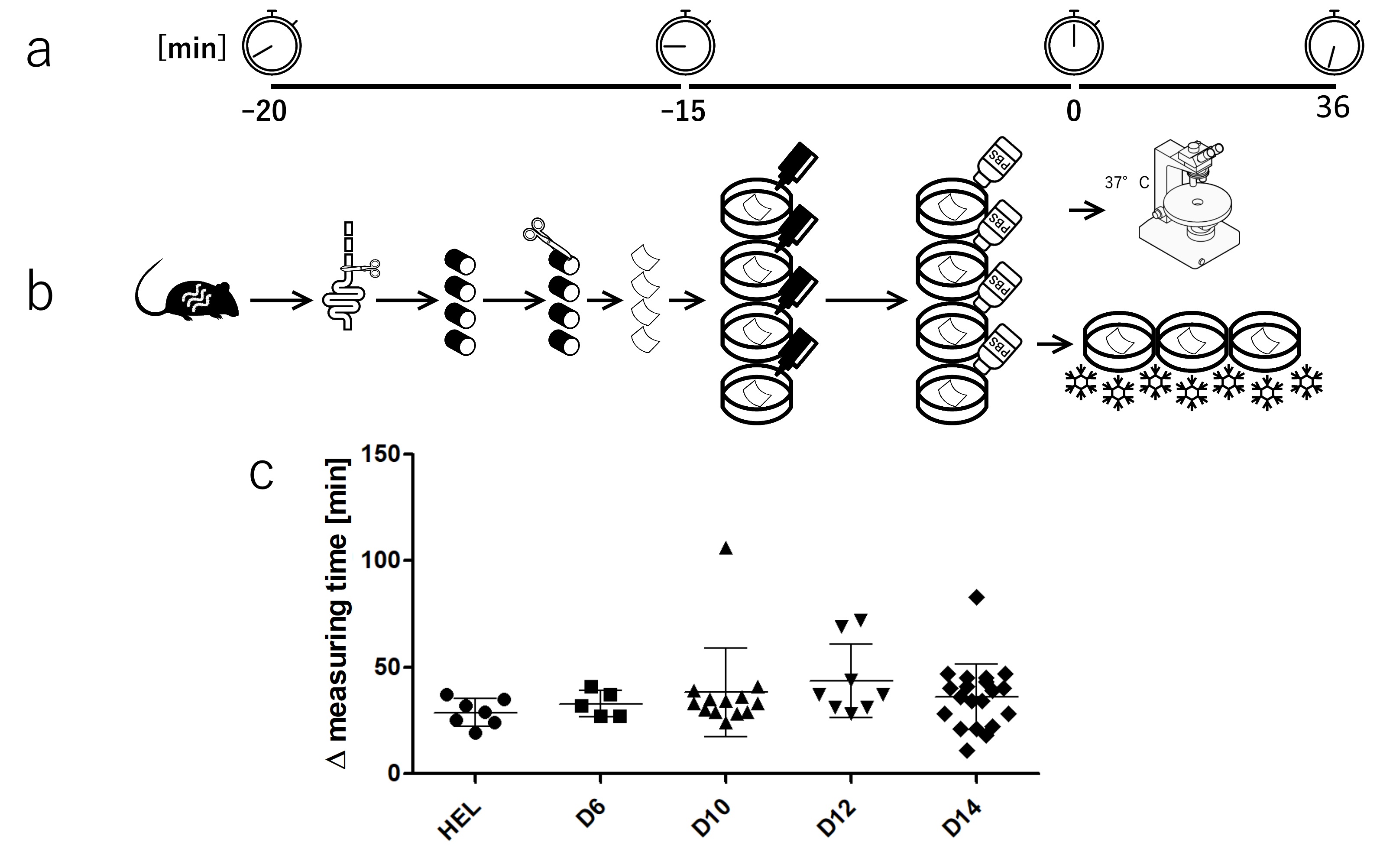

Supplement: Supplementary file 3 — Supplementary Figure 2. [file 41598_2022_10705_MOESM3_ESM.tiff]

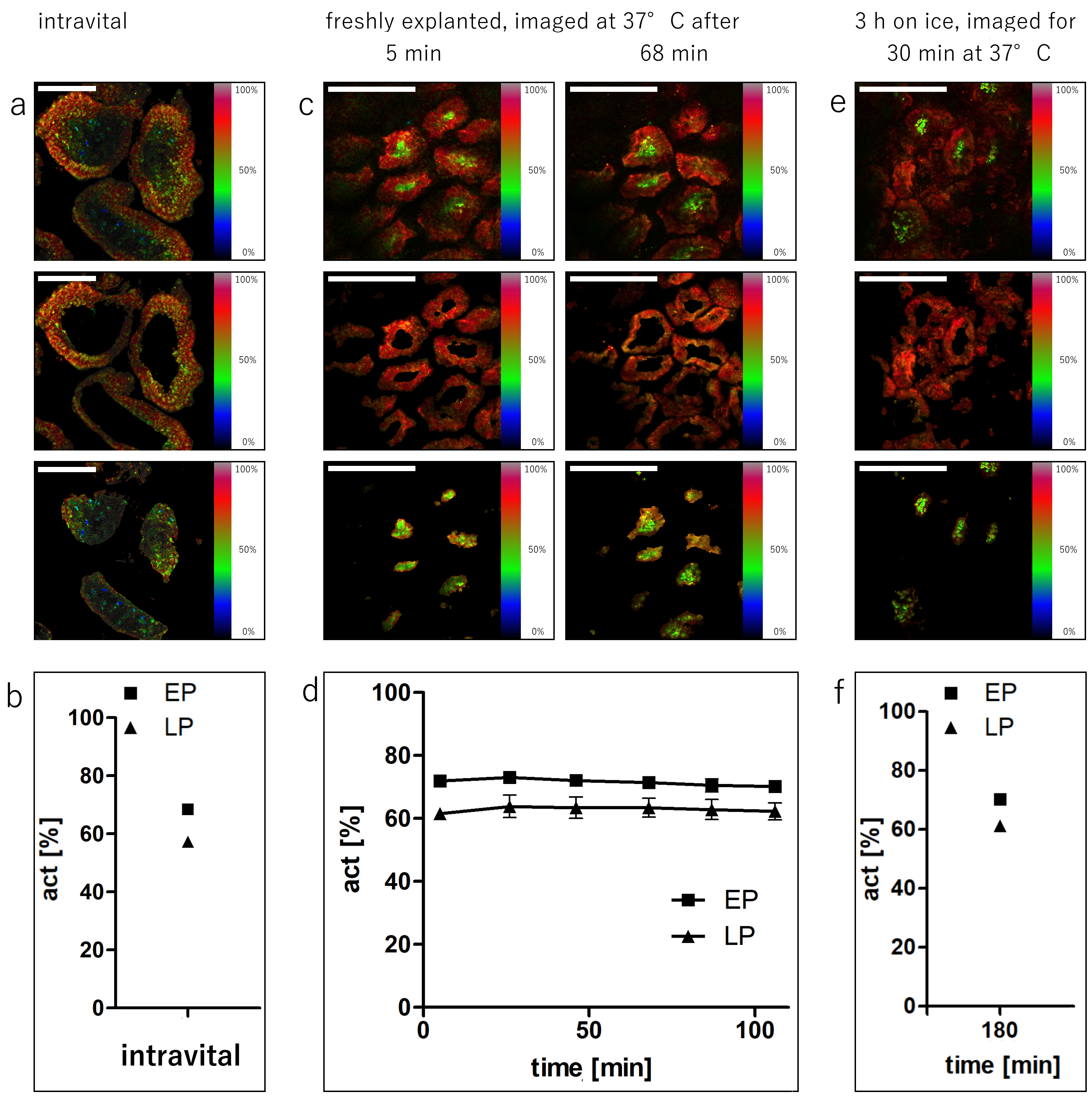

Supplement: Supplementary file 4 — Supplementary Figure 3. [file 41598_2022_10705_MOESM4_ESM.tiff]

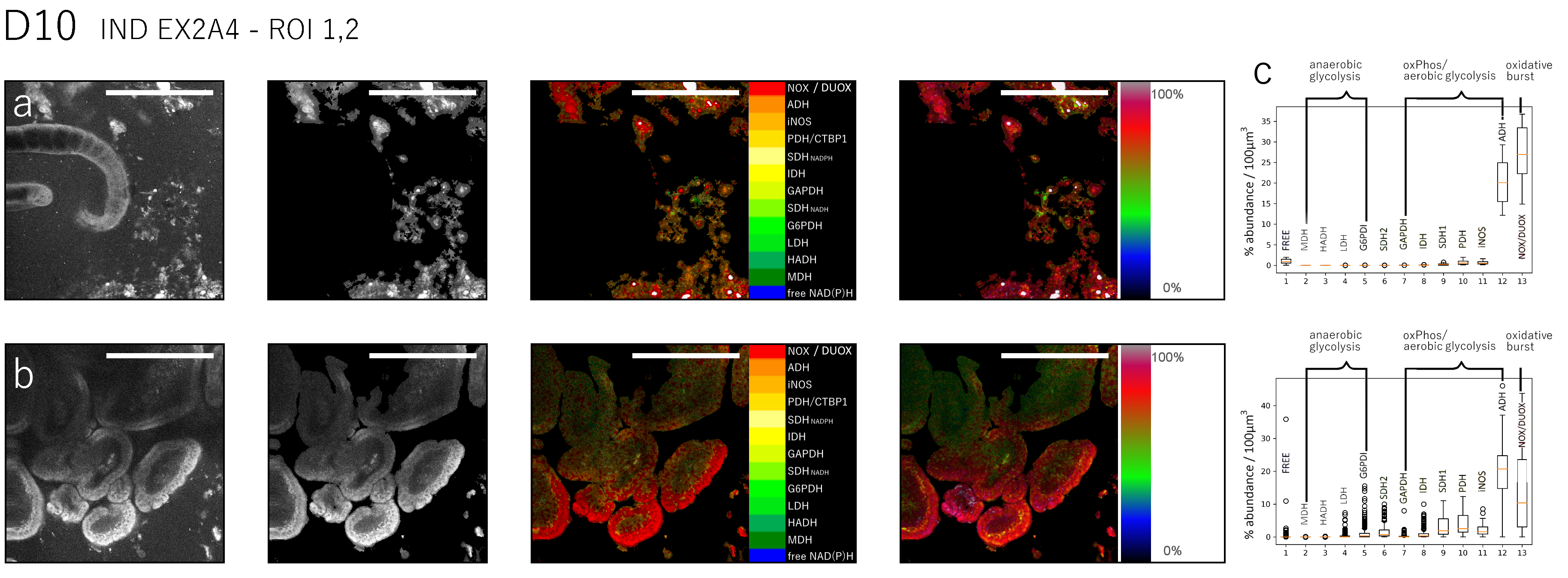

Supplement: Supplementary file 5 — Supplementary Figure 4. [file 41598_2022_10705_MOESM5_ESM.tiff]

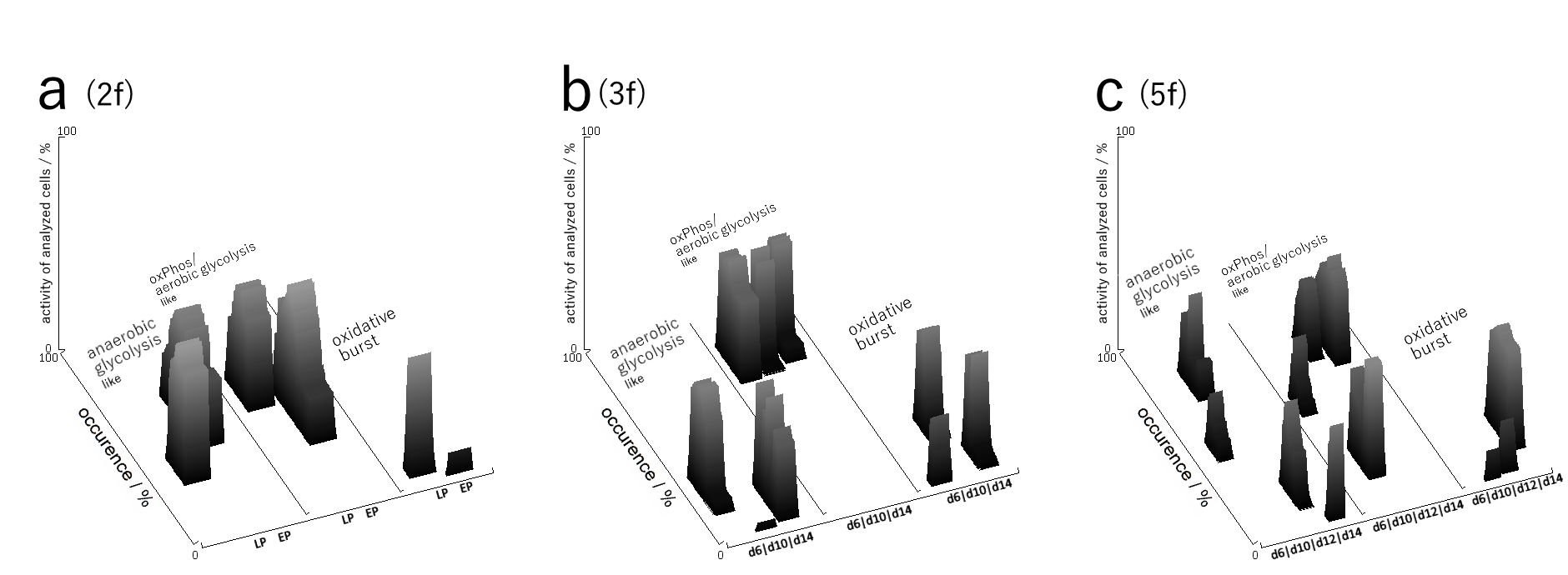

Supplement: Supplementary file 6 — Supplementary Figure 5. [file 41598_2022_10705_MOESM6_ESM.png]
